# Bigleaf—An R package for the calculation of physical and physiological ecosystem properties from eddy covariance data

**DOI:** 10.1371/journal.pone.0201114

**Published:** 2018-08-14

**Authors:** Jürgen Knauer, Tarek S. El-Madany, Sönke Zaehle, Mirco Migliavacca

**Affiliations:** 1 Department of Biogeochemical Integration, Max Planck Institute for Biogeochemistry, Jena, Germany; 2 Michael-Stifel-Center Jena for Data-Driven and Simulation Science, Jena, Germany; Pacific Northwest National Laboratory, UNITED STATES

## Abstract

We present the R package bigleaf (version 0.6.5), an open source toolset for the derivation of meteorological, aerodynamic, and physiological ecosystem properties from eddy covariance (EC) flux observations and concurrent meteorological measurements. A ‘big-leaf’ framework, in which vegetation is represented as a single, uniform layer, is employed to infer bulk ecosystem characteristics top-down from the measured fluxes. Central to the package is the calculation of a bulk surface/canopy conductance (*G*_s_/*G*_c_) and a bulk aerodynamic conductance (*G*_a_), with the latter including formulations for the turbulent and canopy boundary layer components. The derivation of physical land surface characteristics such as surface roughness parameters, wind profile, aerodynamic and radiometric surface temperature, surface vapor pressure deficit (VPD), potential evapotranspiration (ET), imposed and equilibrium ET, as well as vegetation-atmosphere decoupling coefficients, is described. The package further provides calculation routines for physiological ecosytem properties (stomatal slope parameters, stomatal sensitivity to VPD, bulk intercellular CO_2_ concentration, canopy photosynthetic capacity), energy balance characteristics (closure, biochemical energy), ancillary meteorological variables (psychrometric constant, saturation vapor pressure, air density, etc.), customary unit interconversions and data filtering. The target variables can be calculated with a different degree of complexity, depending on the amount of available site-specific information. The utilities of the package are demonstrated for three single-level (above-canopy) eddy covariance sites representing a temperate grassland, a temperate needle-leaf forest, and a Mediterranean evergreen broadleaf forest. The routines are further tested for a two-level EC site (tree and grass layer) located in a Mediterranean oak savanna. The limitations and the ecophysiological interpretation of the derived ecosystem properties are discussed and practical guidelines are given. The package provides the basis for a consistent, physically sound, and reproducible characterization of biometeorological conditions and ecosystem physiology, and is applicable to EC sites across vegetation types and climatic conditions with minimal ancillary data requirements.

## Introduction

The eddy covariance (EC) technique provides direct and continuous measurements of the exchange of heat, water vapor, carbon dioxide, and other trace gases between the surface and the lower atmosphere [1, 2]. The method has significantly contributed to our understanding of how this mass and energy exchange is controlled by environmental drivers such as radiation [3, 4], temperature, vapor pressure deficit (VPD) [5, 6], or soil water stress [7], and how it is modulated by meteorological extreme events such as heatwaves [8, 9]. EC data have proven useful to characterize climate and vegetation controls on the partitioning of available energy at the land surface [10] and the resulting surface hydrology [11]. EC data have further allowed a more detailed insight into the coupling of biogeochemical cycles, in particular carbon and water, and its modification by climate and surface conditions [12, 13].

These findings have been achieved by a large scientific community [14, 15], which maintains several hundred EC measurement sites around the globe. The increasing length of available EC data in combination with freely available data processing tools [16, 17], which are partly available in R [18, 19], underline the important role of EC data in present and future ecological and climate change research.

**Fig 1:**
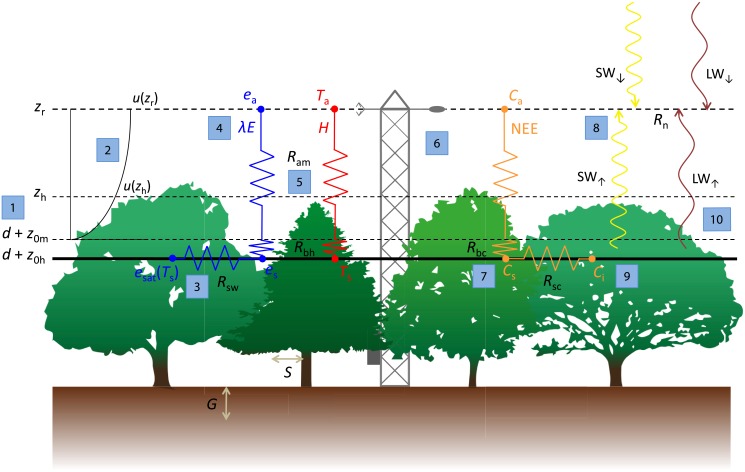
Illustration of the ‘big-leaf’ concept and main functions included in the bigleaf R package. *d* is the displacement height, *z*_0h_ is the roughness length for heat, *z*_0m_ is the roughness length for momentum, *z*_h_ is the average vegetation height, *z*_r_ is the reference (=measurement) height, *u* is the horizontal wind speed, *R*_sw_ is the surface resistance to water vapor, *R*_sc_ is the surface resistance to CO_2_, *R*_bh_ is the canopy boundary layer resistance to heat transfer, *R*_bc_ is the canopy boundary layer resistance to CO_2_ transfer, *R*_am_ is the aerodynamic resistance to momentum transfer, *e*_sat_ is the saturation vapor pressure at the ‘big-leaf’ surface, *e*_s_ is the vapor pressure at the ‘big-leaf’ surface, *e*_a_ is the vapor pressure at reference height, λE is the latent heat flux, *T*_s_ is the aerodynamic surface temperature, *T*_a_ is the air temperature, *H* is the sensible heat flux, *C*_i_ is the bulk intercellular CO_2_ concentration, *C*_s_ is the CO_2_ concentration at the ‘big-leaf’ surface, NEE is the net ecosystem exchange of CO_2_, SW↓ and SW↑ are the incoming and outgoing shortwave radiation, respectively, LW↓ and LW↑ are the incoming and outgoing longwave radiation, respectively, and *R*_n_ is the net radiation. Numbers denote the following functions: 1) roughness.parameters(); 2) stability.parameter(), stability.correction(), wind.profile(); 3) surface.conductance(), stomatal.sensitivity(), stomatal.slope(); 4) potential.ET(), equilibrium.imposed.ET(), WUE.metrics(); 5) aerodynamic.conductance(), decoupling(); 6) energy.closure(); 7) surface.conditions(); 8) light.response(), light.use.efficiency(); 9) intercellular.CO2(), photosynthetic.capacity(), biochemical.energy(), energy.use.efficiency(); 10) radiometric.surface.temp(). For details on the functions, see section ‘Package content’ or the respective R package help pages.

The analysis of EC data does not have to be restricted to direct or partitioned energy and mass flux measurements, but additional ecosystem properties can be derived from a joint analysis of fluxes and meteorological variables. Such additional information can help in obtaining a more comprehensive understanding of the biological and physical processes underlying the measured fluxes (Fig 1). For instance, the aerodynamic conductance (*G*_a_) between the land surface and the instrument height is a key variable describing how effective the ecosystem can transfer mass and energy to the atmosphere. Knowledge of both *G*_a_ and the measured energy or mass fluxes allows to infer average conditions at the surface (e.g. temperature, atmospheric humidity, CO_2_ concentration). This is of interest as conditions at the canopy surface are in general more relevant for ecophysiological processes than those measured at instrument height some distance above the canopy [20].

An important ecophysiological ecosystem property is the surface conductance (*G*_s_). Its vegetation component (canopy conductance (*G*_c_)) is an integrated measure of stomatal conductance and constitutes the main biological control on the exchange of water and carbon dioxide at the land surface. These two central bulk conductances (*G*_a_ and *G*_s_) can be combined to assess the aerodynamic coupling between the vegetation and the atmosphere [21], which again indicates the relative importance of key meterological drivers and the degree of physiological control on evapotranspiration (ET) [21, 22]. Ecosystems well coupled to the atmosphere, such as aerodynamically rough forests, are more likely to exhibit stronger stomatal control on transpiration than low-statured ecosystems such as grasslands [21]. At the same time, ET is under stronger control of VPD in well-coupled ecosystems, whereas available energy has been identified as the decisive factor in poorly coupled ecosystems [22].

The derived *G*_s_ can be used to infer additional ecophysiological variables at ecosystem level such as intrinsic water-use efficiency metrics [23], intercellular CO_2_ concentration (*C*_i_) [24], stomatal sensitivity to VPD [6, 25], or photosynthetic capacity [24, 26]. Many of these quantities can be seen as ecosystem scale analogues of parameters derived from leaf level measurements, and in theory constitute time-invariant quantities that characterize ecosystem functioning in a more comparable manner than flux measurements alone [27].

Since the EC method in its traditional application (i.e. single-level and time-averaged measurements) cannot resolve the vertical and horizontal distribution of ecosystem flux sources and sinks, the above described quantities inevitably lack information on the vertical and horizontal structure of the ecosystem as well as on its components (e.g. soil and vegetation) when they are inferred directly from the measured fluxes. Approaches directed to circumvent this limitation are two-level sensor systems [28, 29], techniques resolving the spatio-temporal variability of the fluxes [30], or the inversion of more detailed models which separate e.g. sunlit from shaded canopy fractions [31, 32], soil from canopy components [33], or which represent the canopy as a multi-layered system [34]. These alternative modeling approaches are able to give more detailed and more realistic insights into the underlying physical and physiological mechanisms. However, the additional complexity comes at the cost of higher computational demands as well as higher requirements on ancillary data for model parameterization. A much simpler and more direct way to infer ecosystem properties from EC data is to invert a ‘big-leaf’ model, in which measured fluxes are assumed to origin from a single, homogenous plane. This approach requires little site-specific ancillary information, is widely applicable across sites, and has been shown to give meaningful results within its limits of applicability and validity [35, 36]. Bulk ecosystem properties derived with a top-down ‘big-leaf’ approach are thus commonly presented in EC studies and have proven useful in characterizing vegetation behavior in various ecosystems and under contrasting conditions [10, 29, 37–41].

Despite their relevance for global change research and their widespread appearance, little effort has been put into the development of harmonized calculation protocols for these quantities, and as a consequence, calculated metrics are often not easily comparable, especially with respect to the wide variety of existing methodologies and formulations (e.g. [42]). In this paper, we describe the R package bigleaf, which provides functions to infer *G*_a_, *G*_s_ and further physical as well as physiological bulk ecosystem properties from EC data and concurrent meteorological measurements in a consistent and standardized manner. In the following, the main equations are presented and their use is demonstrated for four contrasting EC sites. The limitations of the calculations, arising from methodological constraints and inherent limitations of the ‘big-leaf’ approach, as well as the consequences for the interpretation of the resulting variables, are discussed. The paper ends with practical guidelines on how to use the bigleaf package.

## The bigleaf R package

### Package design and availability

The bigleaf package is entirely written in the open source software R [43]. The package is available as a stable version from CRAN (https://cran.r-project.org/web/packages/bigleaf) or as a development version (continously updated with git version control) from http://www.bitbucket.org/juergenknauer/bigleaf. This paper describes package version 0.6.5 (git commit: fcada22). An overview of the main functions is illustrated in Fig 1. In the following, the theory underlying the package’s key functions is shortly presented. For technical details on the functions, the reader is directed to the functions’ help pages and examples therein.

### The ‘big-leaf’ framework

All functions provided in this package are based on the ‘big-leaf’ framework (Fig 1) [44], which assumes that a single plane located at height *d* + *z*_0h_ (*d* = displacement height, *z*_0h_ = roughness length for heat) is the single source and sink of all mass and energy fluxes, and that wind speed is zero at height *d* + *z*_0m_ (*z*_0m_ = roughness length for momentum) and increases exponentially with height. This approach does not distinguish fluxes from different compartments of the ecosystem (e.g. soil and vegetation), nor does it account for vertical variations within the canopy or horizontal heterogeneity due to e.g. different species. The derived quantities at the ‘big-leaf’ surface must thus be regarded as average (but representative) conditions of the tower footprint. The main principle of the bigleaf package is to derive ecosystem surface properties from the observations using a top-down (inversion) approach.

### Package content

#### Data filtering

The bigleaf package does not provide functionalities to pre-process raw EC data or to assess the quality of individual datapoints. Instead, the package relies on correctly pre-processed, aggregated, quality-flagged, and friction velocity (u_*_) filtered fluxes and meteorological measurements (e.g. [19, 45]). Further, some analyses presented in this paper are only meaningful if certain meteorological conditions are met (e.g. daytime or rainfree periods, see below).

The package offers a basic data filtering routine (function filter.data()), which filters EC data based on the aforementioned criteria. The function consists of two parts: 1) Quality control: data points of bad quality (e.g. gap-filled with poor confidence) are discarded, and 2) Meteorological filtering: variables falling out of the (purpose-specific) accepted range (e.g. nighttime values, precipitation events) are filtered out. The filter.data() function returns the input data frame in wich time periods that do not fulfill the filter criteria are set to NA.

#### Constants, unit interconversions, and sign convention

The package combines all required constants into one list that can be evoked by calling bigleaf.constants(). This list is passed as a default argument to all functions that use one or more constants. Thus, individual constants do not have to be provided for any function call, but can be changed by calling the argument explicitly. As a basis for many calculation steps, common unit interconversions are provided:

- Conductances between mass and molar units (m s^−1^ and mol m^−2^ s^−1^)
- Water fluxes between mass and energy units (kg m^−2^ s^−1^ and W m^−2^)
- Carbon fluxes between mass and molar units (g C m^−2^ d^−1^ and μmol CO_2_ m^−2^ s^−1^)
- Atmospheric humidity between vapor pressure deficit (kPa), vapor pressure (kPa), specific humidity (kg kg^−1^), and relative humidity
- Radiation between energy and molar units (W m^−2^ and μmol m^−2^ s^−1^)

The sign convention is that fluxes directed away from the surface are positive and those directed toward the surface are negative. Thus, negative net CO_2_ ecosystem exchange (NEE) values indicate a net uptake of CO_2_ by the ecosystem.

#### Meteorological variables

Most of the central functions in the bigleaf package require meteorological variables that are not commonly provided by the processed EC products, but which can be readily calculated from standard meteorological variables like air temperature, humidity, and atmospheric pressure. For reasons of space, the individual formulations are not presented here, instead the user is directed to the help page of the respective function and the references therein. All functions apply textbook calculations and include:

- latent heat of vaporization: latent.heat.vaporization(*T*_a_)
- psychrometric constant: psychrometric.constant(*T*_a_, *p*)
- saturation vapor pressure and slope of the saturation vapor pressure curve: Esat.slope(*T*_a_)
- air density: air.density(*T*_a_, *p*)
- virtual temperature: virtual.temp(*T*_a_, *q*)
- wet-bulb temperature: wetbulb.temp(*T*_a_, *p*, *D*_a_)
- dew point: dew.point(*T*_a_, *D*_a_)

where *T*_a_ is the air temperature (°C), *p* is the atmospheric pressure (kPa), *q* is the specific humidity (kg kg^−1^), and *D*_a_ is the vapor pressure deficit (kPa). If *p* is not available, it can be approximated by the hypsometric equation as a function of site elevation (pressure.from.elevation()).

#### Aerodynamic conductance

Aerodynamic conductance to heat transfer (*G*_ah_) is central to the ‘big-leaf’ concept and multiple formulations have been proposed. *G*_ah_ can be written as

$$\begin{array}{c} G_{\text{ah}}=1/R_{\text{ah}}={\left(R_{\text{am}}+R_{\text{bh}}\right)}^{-1} \end{array}$$ (1)

where *R*_am_ is the aerodynamic resistance to momentum transfer with turbulence as the principal transport mechanism, and *R*_bh_ is the canopy (quasi-laminar) boundary layer resistance (“excess resistance”) to heat transfer, which is characterized by molecular diffusion as the dominant transport mechanism [46, 47]).

At EC sites, *G*_am_ can be calculated directly as (e.g. [46, 48](aerodynamic.conductance()):

$$\begin{array}{c} G_{\text{am}}=\frac{u_{*}^{2}}{u(z_{\text{r}})} \end{array}$$ (2)

where u_*_ is friction velocity (m s^−1^) and *u*(*z*_r_) is wind speed (m s^−1^) at reference (=measurement height)(m).

Eq 2 implicitly accounts for the effects of atmospheric stability on *G*_am_. Nevertheless, an alternative and frequently used formulation is provided, which explicitly accounts for the effects of atmospheric stability ([46]):

$$\begin{array}{c} G_{\text{am}}=\frac{ku_{*}}{\ln[\frac{z_{\text{r}}-d}{z_{\text{0m}}}]-\psi_{\text{h}}} \end{array}$$ (3)

where *k* is the von Kármán constant (0.41), *d* is the zero plane displacement height (m), *z*_0m_ is the roughness length for momentum (m), and *ψ*_h_ is the integrated form of the stability correction function for heat and water vapor. *ψ*_h_ is a function of the atmospheric stability parameter *ζ* = (*z*_r_ − *d*)/*L*, where *L* is the Monin-Obukhov length. The function stability.correction() can be used to calculate *ψ*_h_ based on formulations suggested by [49] or [50]. The two roughness parameters *d* and *z*_0*m*_ have to be determined a priori. The function roughness.parameters() provides three options: 1) an empirical approach assuming *d* and *z*_0m_ as constant fractions of canopy height *z*_h_ (by default *d* = 0.7*z*_h_ and *z*_0*m*_ = 0.1*z*_h_), 2) a semi-empirical approach estimating both *z*_0m_ and *d* based on *z*_h_ and leaf area index (LAI) according to [51] for data presented in [52], and 3) an approach that calculates *z*_0m_ from the logarithmic wind profile equation with a prescribed *d*. Note that *d* and *z*_0*m*_, as well as all other ancillary variables (e.g. LAI), can be provided as time-varying vectors with the same length as the input data frame.

Multiple formulations have been suggested for the calculation of the canopy (quasi-laminar) boundary layer conductance to heat transfer (*G*_bh_), which range from empirical to physically-based (see [53, 54] for an overview). [55] suggested a simple empirical relationship between *G*_bh_ and u_*_ (Gb.Thom()):

$$\begin{array}{c} G_{\text{bh},\text{Thom}}={\left(6.2u_{*}^{-0.67}\right)}^{-1} \end{array}$$ (4)

Several further (semi-) empirical formulations have been suggested, but we restricted the functions to those best applicable to EC sites. In that respect, relationships based on the Reynolds number, which have been found to show a biphasic behavior [56], are currently not implemented. More mechanistic, but also parameter-rich approaches commonly require LAI and aerodynamically-relevant foliage characteristics (leaf width or leaf characteristic dimension). The formulation suggested by [51] is given by (Gb.Choudhury()):

$$\begin{array}{c} G_{\text{bh},\text{Choudhury}}=\text{LAI}\left(0.02/\alpha \right)\sqrt{u(z_{\text{h}})/w}\left(1-\exp\left(-\alpha /2\right)\right) \end{array}$$ (5)

where *α* is an attenuation coefficient modeled in dependence on LAI according to data presented in [57], *u*(*z*_h_) is wind speed (m s^−1^) at canopy height *z*_h_, and *w* is leaf width (m). Wind speed at height *z*_h_ (or any other height *z* > *d* + *z*_0m_ can be estimated from the logarithmic wind profile equation (wind.profile()):

$$\begin{array}{c} u\left(z\right)=\left(u_{*}/k\right)\ln\left(\left(z-d\right)/z_{\text{0m}}\right)-\psi_{\text{m}} \end{array}$$ (6)

where *ψ*_m_ is the integrated form of the stability correction function for momentum (as calculated in stability.correction()). A third model currently implemented in the bigleaf package was developed by [47] and simplified by [58](Gb.Su()):

$$\begin{array}{c} G_{\text{bh},\text{Su}}=\frac{ku_{*}}{\frac{kC_{\text{d}}f_{\text{c}}^{2}}{4C_{\text{t}}\frac{u_{*}}{u(z_{\text{h}})}}+kB_{s}^{-1}{\left(1-f_{\text{c}}\right)}^{2}} \end{array}$$ (7)

where *C*_d_ is a foliage drag coefficient (assumed constant with a value of 0.2 [47]), *f*_c_ is fractional vegetation cover, *C*_t_ is a heat transfer coefficient, and $B_{s}^{-1}$ is the inverse Stanton number for bare soil surface [58]. *C*_t_ mainly depends on the leaf characteristic dimension and the number of leaf sides participating in heat transfer, see [47] and [58] for details. The denominator of Eq 7 is often referred to as the $kB_{\text{h}}^{-1}$ parameter (e.g. [54]), which is defined as:

$$\begin{array}{c} kB_{\text{h}}^{-1}=\ln(\frac{z_{\text{0m}}}{z_{\text{0h}}})=\frac{ku_{*}}{G_{\text{bh}}} \end{array}$$ (8)

From Eq 8 the roughness length for heat (*z*_0h_) can be determined.

Note that *G*_am_ is identical for different scalars in the atmosphere (heat, water vapor, CO_2_, and other trace gases), whereas *G*_b_ differs with respect to the quantity of interest. *G*_b_ of quantity x can be calculated based on *G*_bh_ [59]:

$$\begin{array}{c} 1/G_{\text{bx}}=1/G_{\text{bh}}\;{\left(\frac{{\text{Sc}}_{x}}{\text{Pr}}\right)}^{0.67} \end{array}$$ (9)

where Pr is the Prandtl number (0.71), and Sc_x_ is the Schmidt number for quantity x. For simplicity, the assumption is made that *G*_b_ is identical for heat and water vapor transfer (i.e. *G*_bh_ = *G*_bw_). The more realistic difference of a few percent [59] is considered small compared to other uncertainties (see also [60]).

Since the calculations of *G*_am_ and *G*_bh_ are independent, the bulk aerodynamic conductance to heat transfer (*G*_ah_) can be calculated as the sum of the inverse versions of Eqs 2, 3 and 4–7. The main function aerodynamic.conductance() returns *G*_am_, *G*_ah_, *G*_bh_, *G*_ac_ (aerodynamic conductance to CO_2_ transfer), *G*_bc_, the corresponding resistances, and $kB_{\text{h}}^{-1}$, *ζ*, as well as *ψ*_h_. If one or more additional Schmidt numbers are provided, *G*_a_ and *G*_b_ are calculated for the respective quantities as well. Due to the modular structure of the functions, each of these components can also be calculated individually.

#### Surface conditions

EC measurements are accompanied by meteorological measurements taken at approximately the same height as the flux measurements, usually several meters above the canopy. If *G*_a_ is determined, the bulk transfer relations can be inverted and solved for the surface variable [23, 29]((surface.conditions())):

$$\begin{array}{c} T_{\text{s}}=T_{\text{a}}+\frac{H}{\rho G_{\text{ah}}c_{p}} \end{array}$$ (10)

$$\begin{array}{c} e_{\text{s}}=e_{\text{a}}+\frac{\lambda E\gamma }{\rho G_{\text{ah}}c_{p}} \end{array}$$ (11)

$$\begin{array}{c} D_{\text{s}}=e_{\text{sat}}\left(T_{\text{s}}\right)-e_{\text{s}} \end{array}$$ (12)

$$\begin{array}{c} C_{\text{s}}=C_{\text{a}}+\frac{\text{NEE}}{G_{\text{ac}}} \end{array}$$ (13)

where *H* is the sensible heat flux (W m^−2^), *ρ* is the air density (kg m^−3^), *c*_*p*_ is the heat capacity of dry air (J K^−1^ kg^−1^), *e* is vapor pressure (kPa), λ*E* is the latent heat flux (W m^−2^), *γ* is the psychrometric constant (kPa K^−1^), *e*_sat_ is the saturation vapor pressure, *D* is the vapor pressure deficit (kPa), and *C* is the CO_2_ concentration. Subscripts a and s denote air and surface, respectively. Note that in Eqs 10–13 “surface conditions” refer to the notional canopy surface. It is also possible to infer conditions in the intercanopy airspace by replacing *G*_ah_ in Eqs 10 and 11 or *G*_ac_ in Eq 13 with *G*_am_. The function surface.conditions() returns *T*_s_, *e*_sat_(*T*_s_), *e*_s_, *D*_s_, *q*_s_, *rH*_s_, and *C*_s_. This method can be applied to other atmospheric constituents measured at EC sites (e.g. methane, nitrogen oxides, ozone), provided that the corresponding *G*_a_ is known (see above).

An alternative estimate of surface temperature is based on the physical principle that any object emits longwave radiation in dependence of its temperature as described by the Stephan-Boltzmann relation. This radiometric surface temperature (*T*_r_, in Kelvin) is given by (e.g. [61], radiometric.surface.temp()):

$$\begin{array}{c} T_{\text{r}}={\left(\frac{{\text{LW}}_{↑}-\left(1-\epsilon \right){\text{LW}}_{↓}}{\sigma \epsilon }\right)}^{1/4} \end{array}$$ (14)

where LW_↑_ and LW_↓_ are longwave upward and longwave downward radiation (W m^−2^), respectively, *σ* is the Stefan-Boltzmann constant (W m^−2^ K^−4^), and *ϵ* is the emissivity of the surface.

#### Surface conductance

Surface conductance to water vapor (*G*_sw_ in m s^−1^), describes the conductance of the entire surface, i.e. including soil and plant canopy components. It is commonly calculated by inverting the Penman-Monteith (PM) equation (surface.conductance()):

$$\begin{array}{c} G_{\text{sw}}=\frac{\lambda EG_{\text{ah}}\gamma }{s\left(R_{\text{n}}-G-S\right)+\rho c_{p}G_{\text{ah}}D_{\text{a}}-\lambda E\left(s+\gamma \right)} \end{array}$$ (15)

where *s* is the slope of the saturation vapor pressure curve (kPa K^−1^), *R*_n_ is the net radiation (W m^−2^), *G* is the ground heat flux (W m^−2^), and *S* is the sum of all energy storage fluxes (W m^−2^).

Eq 15 implicitly assumes that *G*_a_ for water vapor equals *G*_a_ for heat, i.e. *G*_ah_ = *G*_aw_ which corresponds to an amphistomatous vegetation where the transfer of both heat and water vapor occurs at both leaf sides. The hypostomatous case (water vapor transfer from one side only) is conceptually not straightforward at the canopy level [22, 42], and is thus currently not implemented in this package. Eq 15 further assumes that the energy balance is closed (i.e. *R*_n_ − *G* − *S* = λ*E* + *H*). The derived *G*_sw_ and all subsequent derivations are sensitive to violations of this assumption [23, 62]. The function surface.conductance() offers the calculation of *G*_sw_ according to Eq 15, and a simplified (but also less realistic) formulation based on a simple flux-gradient approach, which assumes infinite *G*_ah_: *G*_sw_ = λ*E*/λ/(*D*_a_/*p*). This formulation is equivalent to the one proposed by [63].

#### Vegetation-atmosphere decoupling

With both *G*_ah_ and *G*_sw_ available, the degree of aerodynamic decoupling between the land surface and the atmosphere can be assessed with the decoupling coefficient *Ω*, which takes values between 0 and 1. Low values indicate well-coupled conditions and a high degree of physiological control on ET. Values close to 1 indicate the opposite, i.e. poorly coupled conditions and a low sensitivity of ET to *G*_sw_ [21, 22]. In its simplest and most commonly used form, *Ω* is given by [21] (decoupling()):

$$\begin{array}{c} \Omega_{\text{Jarvis}}=\frac{s/\gamma +1}{s/\gamma +1+G_{\text{ah}}/G_{\text{sw}}} \end{array}$$ (16)

Eq 16 was modified by [64], who included the effects of radiative coupling between the vegetation and the atmosphere:

$$\begin{array}{c} \Omega_{\text{Martin}}=\frac{s/\gamma +1+G_{\text{r}}/G_{\text{ah}}}{s/\gamma +1+G_{\text{ah}}/G_{\text{sw}}+G_{\text{r}}/G_{\text{sw}}+G_{\text{r}}/G_{\text{ah}}} \end{array}$$ (17)

where *G*_r_ is the longwave radiative transfer conductance of the canopy (m s^−1^), calculated as $G_{\text{r}}=4\sigma T_{\text{a}}^{3}\text{LAI}/c_{p}$ (longwave.conductance()). Note that, as in the PM equation (Eq 15), Eqs 16 and 17 assume that the vegetation is amphistomatous [21].

#### Imposed and equilibrium evapotranspiration

The concept of decoupling is often used to characterize physiological and energy controls on transpiration. In addition it can help to quantify radiation and VPD controls on λE (e.g. [65]). λE can be written in an alternative way [21](equilibrium.imposed.ET()):

$$\begin{array}{c} \lambda E=\Omega \lambda E_{\text{eq}}+\left(1-\Omega \right)\lambda E_{\text{imp}} \end{array}$$ (18)

where

$$\begin{array}{c} \lambda E_{\text{eq}}=\frac{s(R_{\text{n}}-G-S)}{s+\gamma } \end{array}$$ (19)

and

$$\begin{array}{c} \lambda E_{\text{imp}}=\frac{\rho c_{p}G_{\text{sw}}D_{\text{a}}}{\gamma } \end{array}$$ (20)

Eqs 19 and 20 are derived directly from the PM equation by letting *G*_ah_ approach 0 or ∞, respectively. Thus, λ*E*_eq_ is the λ*E* rate that would occur if the surface was completely decoupled from the atmosphere. In this case, λ*E* is strongly controlled by *R*_*n*_. Likewise, λ*E*_imp_ can be interpreted as the λ*E* rate that would occur under fully coupled conditions, in which case λ*E* is mainly dependent on *G*_sw_ and *D*_a_.

#### Potential evapotranspiration

Potential evapotranspiration (λ*E*_pot_) is frequently used to characterize atmospheric demand and the degree of climatic aridity (e.g. [11]). Here, λ*E*_pot_ is by default calculated from the Priestley-Taylor equation [66] (potential.ET()):

$$\begin{array}{c} \lambda E_{\text{pot},\text{PT}}=\frac{\alpha s\left(R_{\text{n}}-G-S\right)}{s+\gamma } \end{array}$$ (21)

where *α* is the Priestley-Taylor coefficient, which accounts for large-scale advection effects. Its value is usually set to 1.26, but it likely varies with surface conditions [67]). λ*E*_pot_ can further be calculated from the PM equation with a prescribed *G*_sw_ [6], which may correspond to typical maximum values (e.g. 95% quantile) found in the ecosystem:

$$\begin{array}{c} \lambda E_{\text{pot},\text{PM}}=\frac{s\left(R_{\text{n}}-G-S\right)+\rho c_{p}D_{\text{a}}G_{\text{ah}}}{s+\gamma (1+G_{\text{ah}}/G_{\text{sw}})} \end{array}$$ (22)

#### Energy balance

The package contains basic functionalities to characterize energy balance closure at EC sites. The function energy.closure() quantifies the energy balance closure (*R*_n_ − *G* − *S* = λ*E* + *H*) with both the slope method and the energy balance ratio (EBR) as described in [68]. The package further enables the calculation of biochemical energy (*S*_p_), a small and therefore often neglected component of the energy balance: *S*_p_ = *α*NEE, where *α* = 0.422 J mol^−1^ denotes the biochemical energy taken up/released by photosynthesis/respiration per mole of CO_2_ fixed/respired [69]. The function energy.use.efficiency() provides a simple estimate of the energy use efficiency (EUE) of the ecosystem: EUE = *S*_p_/*R*_n_.

#### Physiological ecosystem quantities

For ecosystems that have a largely closed vegetation cover, and under conditions when canopy and soil surfaces are not wet, the derived *G*_s_ can be interpreted as a proxy for the canopy-integrated stomatal conductance (i.e. canopy conductance *G*_c_) [36]. *G*_s_ may then be used to calculate additional physiological quantities. The function stomatal.slope() returns an estimate of the stomatal slope parameter *G*_1_ at ecosystem level, analogous to *g*_1_ at leaf level [41] (Note that in this paper, uppercase and lowercase letters denote physiological quantities at ecosystem and leaf-level, respectively). *G*_1_ is estimated using non-linear regression from the unified stomatal model (USO) [70]:

$$\begin{array}{c} G_{\text{sw}}=G_{0}+1.6(1+\frac{G_{1,\text{USO}}}{\sqrt{D_{\text{s}}}})\frac{\text{GPP}}{C_{\text{s}}} \end{array}$$ (23)

where *G*_0_ is the minimum canopy conductance (mol m^−2^ s^−1^), and GPP is gross primary productivity (μmol CO_2_ m^−2^ s^−1^). *D*_s_ and *C*_s_ represent conditions at the notional ‘big-leaf’ surface in this case (Eqs 12 and 13, respectively), but they are often replaced by the measured values at instrument height (i.e. *D*_a_ and *C*_a_ [41]. *G*_0_ can either be estimated along with *G*_1_, or fixed to a user-defined value (e.g. set to 0). In addition to Eq 23, *G*_1_ can be calculated from the stomatal model proposed by [71], or from its modified version suggested by [72]. Note that absolute values and units of *G*_1_ differ across models. GPP is not directly measured at EC sites but inferred from NEE-partitioning algorithms (e.g. [73, 74]). GPP is further not directly analogous to leaf-level net photosynthesis (*A*_n_), and ecosystem leaf day respiration, if available, may be subtracted from GPP to better represent canopy-level *A*_n_ [24, 75].

The package further includes several alternative water-use efficiency (WUE) metrics (WUE.metrics()) which can be calculated more readily from the measured fluxes, but which contain less physiological information [23]. Examples are WUE (= GPP/ET), inherent WUE (IWUE = (GPP *D*_a_)/ET) [12], or underlying WUE ($\text{uWUE}=\left(\text{GPP}\sqrt{D_{\text{a}}}\right)/\text{ET}$)/ET) [13].

Stomatal sensitivity to VPD, a relevant indicator of vegetation water-use strategy, can be characterized with the following function [76] (stomatal.sensitivity()):

$$\begin{array}{c} G_{\text{sw}}=-m\ln\left(D_{\text{s}}\right)+b \end{array}$$ (24)

where the two parameters *m* (mol m^−2^ s^−1^ ln(kPa)^−1^) and *b* (mol m^−2^ s^−1^) represent the sensitivity of *G*_sw_ to *D*_s_ (*D*_a_ can be used alternatively) and the reference *G*_sw_ at *D*_s_ of 1 kPa, respectively [6, 76].

Bulk canopy intercellular CO_2_ concentration (*C*_i_ in μmol mol^−1^) can be inferred from Fick’s first law analogously to the calculation of *c*_i_ at leaf level (see e.g [24, 77], intercellular.CO2()):

$$\begin{array}{c} C_{\text{i}}=C_{\text{s}}-\text{GPP}/G_{\text{sc}} \end{array}$$ (25)

where *C*_s_ is the CO_2_ concentration at the ‘big-leaf’ surface (μmol mol^−1^; Eq 13), which can also be approximated by *C*_a_. *G*_sc_ denotes the surface conductance to CO_2_ (mol CO_2_ m^−2^ s^−1^) and is calculated as *G*_sc_ = *G*_sw_/1.6.

With *C*_i_ available, the ‘big-leaf’ concept may be further expanded to calculate an estimate of basic photosynthetic parameters such as the maximum carboxylation rate (*V*_cmax_) and maximum electron transport rate (*J*_max_) at canopy level (e.g. [24, 26, 78], photosynthetic.capacity()). The calculation is once more analogous to that at leaf level, where commonly the model developed by [79] is employed. Note however, that especially for *V*_cmax_ and *J*_max_ the interpretation differs from that at leaf level (see Discussion). From the Rubisco-limited photosynthesis rate (when carboxylation is the rate limiting process i.e. GPP = GPP_c_, usually under high radiation), *V*_cmax_ (μmol m^−2^ s^−1^) can be calculated as:

$$\begin{array}{c} V_{\text{cmax}}=\frac{{\text{GPP}}_{\text{c}}\left(C_{\text{i}}+K_{\text{c}}\left(1+O_{\text{i}}/K_{\text{o}}\right)\right)}{C_{\text{i}}-\Gamma^{*}} \end{array}$$ (26)

where *K*_c_ (μmol mol^−1^) and *K*_o_ (mmol mol^−1^) are the Michaelis-Menten constants for CO_2_ and O_2_, respectively, *O*_i_ (mol mol^−1^) is the O_2_ concentration, and Γ* (μmol mol^−1^) is the photorespiratory CO_2_ compensation point (μmol mol^−1^). All photosynthetic parameters and their temperature responses (activation energies) are taken from [80] and assume infinite mesophyll conductance to CO_2_ transfer. Under conditions when Ribulose 1,5-bisphosphate (RuBP)-regeneration is limiting photosynthesis (i.e. GPP = GPP_j_), the electron transport rate *J* (μmol m^−2^ s^−1^) is given by:

$$\begin{array}{c} J=\frac{{\text{GPP}}_{\text{j}}\left(4C_{\text{i}}+8\Gamma^{*}\right)}{C_{\text{i}}-\Gamma^{*}} \end{array}$$ (27)

*J*_max_ is then calculated from the following relation:

$$\begin{array}{c} J=\frac{{\text{APPFD}}_{\text{PSII}}+J_{\text{max}}-\sqrt{{\left({\text{APPFD}}_{\text{PSII}}+J_{\text{max}}\right)}^{2}-4\Theta {\text{APPFD}}_{\text{PSII}}J_{\text{max}}}}{2\Theta } \end{array}$$ (28)

where APPFD_PSII_ is absorbed photosynthetic photon flux density (PPFD) by photosystem II (μmol m^−2^ s^−1^), and Θ is a curvature parameter. APPFD_PSII_ is currently assumed to be a constant fraction of PPFD (by default APPFD_PSII_ = 0.8PPFD), but a more realistic estimate of APPFD, depending on solar elevation angle and LAI, will be implemented in the future. Bulk canopy photosynthesis is assumed to be limited by either Rubisco activity (GPP = GPP_c_) or RuBP-regeneration (GPP = GPP_j_) at high and low radiation, respectively, and simple radiation thresholds are applied to separate the two limitation states. *V*_cmax_ and *J*_max_ are temperature-dependent and are normalized to the reference temperature of 25°C (i.e. *V*_cmax,25_ and *J*_max,25_) using a modified Arrhenius equation as described in e.g. [81] with default parameter values from [80] and [82].

Ecosystem light response curves (LRCs) are useful to characterize both the CO_2_ uptake rate at light saturation as well as the light utilization efficiency (i.e. the initial slope). The most frequently used model is the rectangular hyperbolic LRC, which can be written in a general form as [83] (light.response()):

$$\begin{array}{c} \text{-NEE}=\frac{\alpha \text{PPFD}}{\left(1-\left(\text{PPFD}/{\text{PPFD}}_{\text{ref}}\right)+\left(\alpha \text{PPFD}/{\text{GPP}}_{\text{ref}}\right)\right)}-R_{\text{eco}} \end{array}$$ (29)

where *α* is the initial slope of the light-response curve (μmol CO_2_ m^−2^ s^−1^ (μmol quanta m^−2^ s^−1^)^−1^), *R*_eco_ is ecosystem respiration (μmol CO_2_ m^−2^ s^−1^), and PPFD_ref_ is the PPFD value at which GPP_ref_ (μmol CO_2_ m^−2^ s^−1^) is calculated (usually at saturating light, e.g. at 2000 μmol m^−2^ s^−1^). Additionally, a simple light-use efficiency (LUE) metric, defined as the ratio of cumulative GPP to cumulative PPFD, is available in the package (light.use.efficiency()).

## Case studies

### Single-level EC sites

Three sites with EC measurements at a single level above the canopy were chosen for the demonstration of the formulations described above: AT-Neu (Neustift), a managed grassland in Austria [84], DE-Tha (Tharandt), a high-statured (mean canopy height = 26.5m) spruce forest in Eastern Germany [85], and FR-Pue (Puechabon), a Mediterranean evergreen oak forest in southern France, which is subject to seasonal water stress [86]. The location as well as basic ecosystem properties for these sites are listed in Table 1. Data are freely available from the FLUXNET2015 dataset (http://fluxnet.fluxdata.org/data/fluxnet2015-dataset/; accessed on 2016-11-09). Subsetted dataframes are included in the package and are automatically loaded when the package is attached. Data underwent standard postprocessing (e.g u_*_ filtering, gap-filling, NEE-partitioning) as detailed on the FLUXNET2015 webpage (http://fluxnet.fluxdata.org/data/fluxnet2015-dataset/data-processing/; accessed on 2018-04-19).

**Table 1:** Characteristics of the three single-level case study sites.

site	lon (°)	lat (°)	elevation (m)	MAP (mm)	MAT (°C)	vegetation type	*z*_h_ (m)	max. LAI
AT-Neu	11.32	47.12	970	852	6.30	grassland	0.5^a^	6^a^
DE-Tha	13.57	50.96	385	843	8.20	spruce forest	26.5	7.6
FR-Pue	3.60	43.74	48	883	13.50	holm oak forest	5.5	3.3

^a^ highly variable throughout the growing season [84]. LAI = 5 in subsequent calculations

#### Seasonal courses of *G*_s_, *G*_a_ and vegetation-atmosphere decoupling

**Fig 2:**
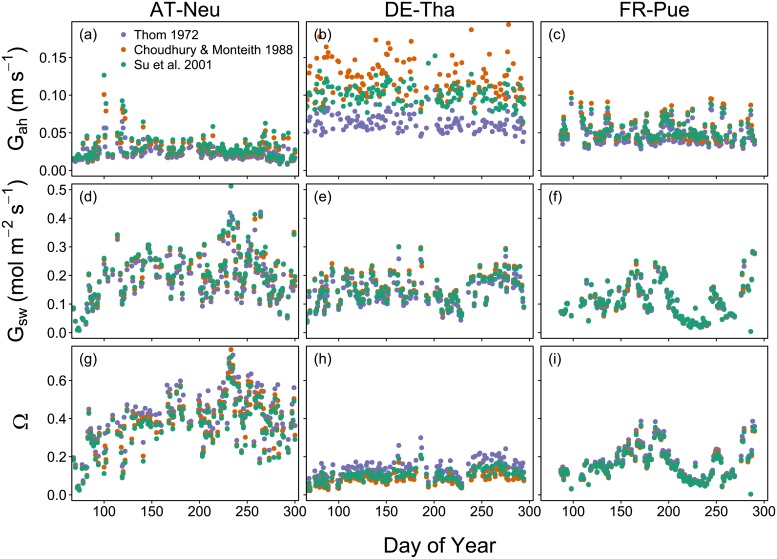
Seasonal courses of mean daily values of aerodynamic conductance to heat transfer (*G*_ah_), surface conductance to water vapor (*G*_sw_), and decoupling coefficient (Ω) for the year 2012. Data were filtered for rainfree periods (24h after rainfall excluded), daylight (PPFD > 200 μmol m^−2^ s^−1^), and positive λE. *G*_sw_ was calculated according to Eq 15, and Ω according to Eq 16. Three different *G*_ah_ formulations (Eqs 2 and 4–7), denoted by different colors, were used as input variables for the respective functions.

We calculated seasonal dynamics of aerodynamic and surface conductance to water vapor, as well as the decoupling coefficient Ω (Fig 2). The results reveal that *G*_ah_ is relatively constant over the course of the year, but differs in magnitude across sites. As expected, highest values can be found in the aerodynamically rough spruce forest DE-Tha, and lowest values in the meadow AT-Neu. FR-Pue shows intermediate values. Differences between the different *G*_ah_ versions result from different models of the bulk boundary layer conductance (*G*_bh_; Eqs 4–7). The different *G*_bh_ formulations agree well for AT-Neu and FR-Pue, but lead to clear differences in estimated *G*_ah_ for DE-Tha. This is likely because the Choudhury (Eq 5) and Su (Eq 7) models consider additional aerodynamic properties (e.g. leaf size, LAI) that are neglected in the Thom model (Eq 4). Thus, accounting for the low leaf characteristic dimension / leaf width and high LAI in DE-Tha leads to a higher *G*_ah_ in the Su and especially in the Choudhury formulation compared to the Thom model. The differences in *G*_ah_ among the formulations do not have strong effects on the derived *G*_sw_ and Ω. *G*_sw_ shows pronounced seasonal dynamics at all three sites. Lowest values correspond to inactive vegetation, as e.g. caused by soil water stress (DOY 190-240 in FR-Pue). The dynamics in *G*_sw_ are clearly reflected in Ω, the magnitude of which differs considerably across sites. AT-Neu (grassland) is relatively poorly coupled, whereas DE-Tha (forest) shows a high degree of coupling. All three sites show typical values for the respective vegetation type [87].

#### Surface conditions

**Fig 3:**
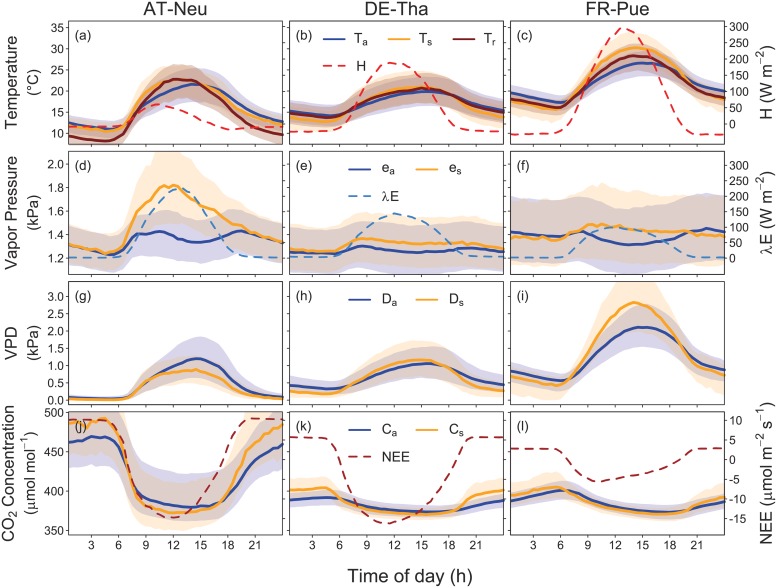
Median diurnal courses of measured air and respective derived ‘big-leaf’ surface variables for the summer months of all available site years (JJA). Lines depict median diurnal courses of all available site years and shaded areas the interquartile range. Surface conditions were calculated with *G*_*a*_ calculated from Eqs 2 and 7 (with *D*_l_ taken as 0.02, 0.008, and 0.035 m for AT-Neu, DE-Tha, and FR-Pue, respectively). Radiometric surface temperature in panels a-c was calculated according to Eq 14 assuming a constant longwave emissivity of 0.98.

Fig 3 depicts mean diurnal courses of air temperature, vapor pressure, VPD, and CO_2_ concentration and the respective surface variables as calculated from Eqs 10–13 for the summer months June, July, and August (JJA) of all available site years. At all three sites, aerodynamic surface temperature *T*_s_ (Eq 10) exceeds air temperature at daytime and is lower at nighttime. *T*_s_—*T*_a_ is largely parallel to the course of *H* throughout the day. The inferred temperature difference depends not only on the magnitude of *H*, but also on *G*_ah_. It follows that the grassland AT-Neu has a more pronounced temperature difference for the same *H* than the forest DE-Tha owing to its lower efficiency to transfer heat to the atmosphere (i.e. lower *G*_ah_). Temperature gradients are most pronounced at FR-Pue (approx. 4°C at midday) where a large fraction of the available energy goes into *H*. Radiometric surface temperature (*T*_r_; Eq 14) generally agrees well with *T*_s_, but shows biases at some timeperiods (e.g. AT-Neu at night). Differences between *T*_s_ and *T*_r_ can be caused by inappropriate emissivity values, biases in the estimated *G*_ah_, or differences in the spatial representativeness of radiation (LW_↑_) and flux (*H*) measurements.

The derived vapor pressure at the ‘big-leaf’ surface (*e*_s_) exceeds the measured values at instrument height (*e*_a_) at all three sites during daytime. The water vapor gradient at AT-Neu is significantly higher than at the other two sites, which is caused by the relatively high λ*E* and low *G*_ah_. The high *e*_s_ at AT-Neu leads to a decrease of surface VPD (*D*_s_) compared to air VPD (*D*_a_). In contrast, the temperature effect on VPD is stronger than the moisture effect in DE-Tha and FR-Pue, with the consequence that *D*_s_ exceeds *D*_a_ at daytime at these two sites. Future analyses should be directed to the question whether these patterns hold across sites and vegetation types.

The difference of CO_2_ concentration at the ‘big-leaf’ surface (*C*_s_) to the concentration in the atmosphere (*C*_a_) follows the diurnal pattern of NEE (Fig 3j–3l). Daytime photosynthetic CO_2_ uptake and nocturnal ecosystem respiration lead to lower or higher CO_2_ concentrations, respectively, at the surface compared to the air. The absolute differences are generally low (< 10 μmol mol^−1^), but may exceed 20 μmol mol^−1^ under conditions of high biological activity and low turbulent mixing.

#### Relationship between *G*_s_ and GPP

**Fig 4:**
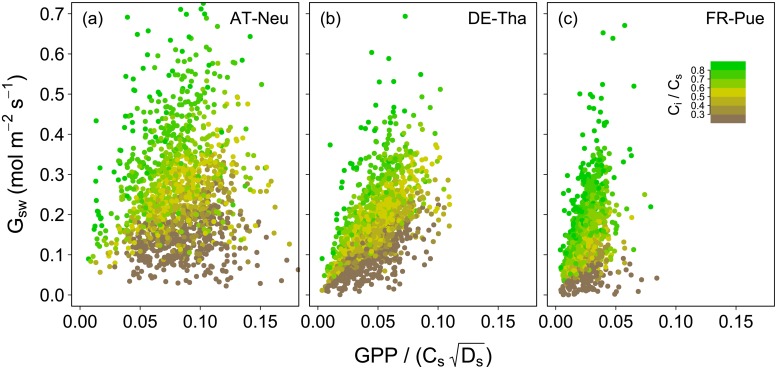
Surface conductance (*G*_sw_) plotted against $\text{GPP}/(C_{\text{s}}\sqrt{D_{\text{s}}})$. The slope of the relationship corresponds approximately to the *G*_1,USO_ parameter (Eq 23). Different colors denote the ratio of bulk intercellular CO_2_ concentration (*C*_i_; Eq 25) to ‘big-leaf’ surface CO_2_ concentration (*C*_s_; Eq 13). Shown are data for rainfree periods in the growing season of 2012 (see text for details on data filtering).

Fig 4 illustrates the relationship between *G*_sw_ and the “stomatal index”, i.e. GPP adjusted for VPD and CO_2_ concentration [41] for the year 2012. The relationship between these two quantities characterizes intrinsic WUE (iWUE) at ecosystem level and provides essential information on the physiological basis of ecosystem WUE. The slope of the depicted relationship approximates the *G*_1,USO_ parameter (“stomatal slope”) with higher slopes corresponding to a lower iWUE. Points in Fig 4 are colored according to the *C*_i_/*C*_s_ ratio, which is again closely related to iWUE. High *C*_i_/*C*_s_ correspond to high stomatal slopes and lower WUE, and the opposite is the case for low *C*_i_/*C*_s_. The relationship between *G*_sw_ and the “stomatal index” shows large scatter, especially at AT-Neu, which indicates variations of iWUE throughout the growing season. Such variations within one year may be caused by changes in phenology, LAI (as e.g. caused by mowing) or the onset of water stress.

### Two-level EC site

The package was further applied to data from the site ES-LMa (Majadas de Tietar), where fluxes and meteorology were measured at two different heights. The site (39°56’N; 5°46’W, 260 m a.s.l.) is an open woodland with a tree canopy cover (mainly *Quercus ilex*) of about 20% [88]. Ecosystem fluxes were measured at 15.5 m above ground (7 m above tree canopy height) and grass layer fluxes were measured with a second tower at 1.65 m height. Tree fluxes were derived as the differences of the ecosystem fluxes and the grass layer fluxes similar to [28, 29].

*G*_1,USO_ and uWUE were calculated for a moving window of +/- 3 weeks which was shifted by one week for each calculation. This procedure was done for the ecosystem, grass layer and trees. Minimum, maximum and mean of mean daily air temperature and soil water content were calculated for the same period.

**Fig 5:**
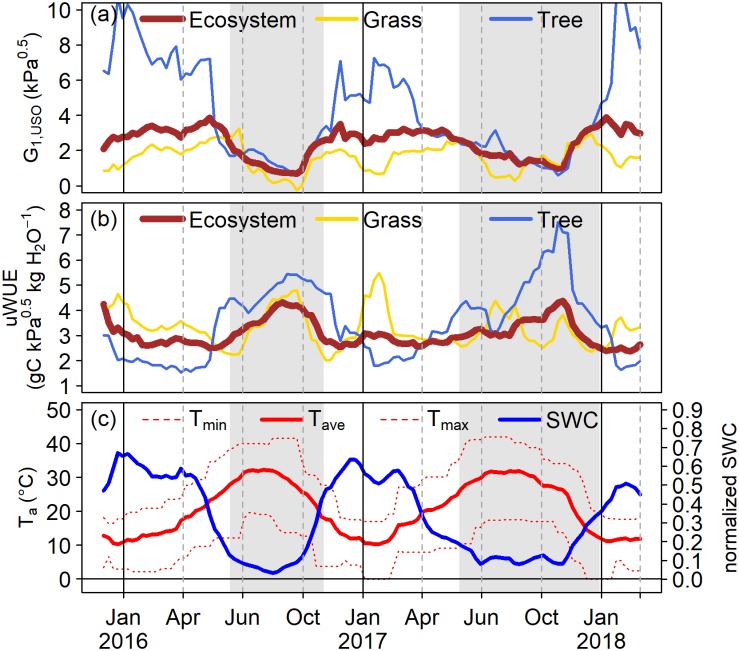
(a) Time series of the stomatal slope parameter *G*_1,USO_ and (b) underlying water-use efficiency (uWUE) calculated for the whole ecosystem (brown), the grass layer (yellow) and the trees (blue) between December 2015 and March 2018. (c) Time series of minimum, maximum and mean daily air temperature and normalized soil water content for the same period. Grey shaded areas denote dry periods associated with a wilted grass layer.

Differences in *G*_1,USO_ follow clear seasonal patterns (Fig 5) depending on water availability, VPD (which follows air temperature), and the associated growth and senescence of the grass layer. Ecosystem *G*_1,USO_ is relatively constant during the growing periods of 2016 and 2017 (winter and spring). *G*_1,USO_ of the grass layer is more variable as compared to the ecosystem. This mirrors the seasonal dynamics and fast responses of the grass layer to environmental conditions. For *G*_1,USO_ of the grass layer a pronounced increase is visible before *G*_1,USO_ drops during the summer drought. The increase is due to the rapid drop in GPP as the grasses start wilting due to drying of the top soil, while λE reduces much slower due to soil evaporation from deeper layers. The subsequent drop in *G*_1,USO_ is then caused by the continuous reduction in λE during the dry period as the deeper soil layers are also drying out. *Q. ilex* trees are rather isohydric and react to increasing VPD by closing their stomata to reduce water losses, which results in a decreasing *G*_1,USO_. In 2017, *G*_1,USO_ of the trees decreases more slowly compared to 2016, which is most likely caused by several rain pulses that increased the water availability and reduced VPD as compared to the long lasting dry period in 2016. *G*_1,USO_ (Fig 5a) and the uWUE (Fig 5b) show strongly anti-correlated patterns. As *G*_1,USO_ increases the uWUE reduces and vice versa. The trees are able to strongly increase their uWUE as atmospheric humidity and soil water availability are reduced.

### Calculated ecosystem characteristics

Tables 2 and 3 present physical and physiological ecosystem properties, respectively, of the four study sites. All quantities represent median growing season values of multiple site years, i.e. have to be interpreted as multi-year averages. Site years used for the calculations were 2002-2012 for AT-Neu, 1996-2014 for DE-Tha, 2000-2014 for FR-Pue, and November 2015—November 2017 for ES-LMa. Growing season was delineated using filter.data() with tGPP = 0.5, ws = 15, min.int = 5 (relative GPP threshold, window size (days), minimum interval (days)). Data were filtered using site-specific, multi-year averaged u_*_ thresholds, daytime conditions (PPFD > 200 μmol m^−2^ s^−1^), and rainfree periods (24h after rainfall excluded). Data were further filtered for *D*_a_ > 0.01 kPa, λ*E* > 0 W m^−2^ and *T*_a_ > 5C. *G*_ah_ was calculated according to Eqs 2 and 7, unless stated otherwise. More information on the ancillary data used for the calculations can be found under http://www.bitbucket.org/juergenknauer/bigleaf/src/master/ancillary. Note that for this study, ancillary variables (e.g. LAI, *z*_h_, *z*_r_) were assumed to be constant throughout all site years. In many cases, however, they vary across the growing season or among years. Thus, for a more realistic representation of the calculated ecosystem properties, required ancillary variables, if available, should be provided at an adequate temporal resolution. In general, computations in the bigleaf package are fast, e.g. with a state-of-the-art PC it takes < 0.1 seconds to calculate *G*_s_ for 10 site years and 2-3 seconds to calculate all properties as shown in Tables 2 and 3.

**Table 2:** Median daytime physical ecosystem properties in the growing season calculated with the bigleaf package.

	AT-Neu	DE-Tha	FR-Pue	ES-LMa	ES-LMa_grass_	ES-LMa_trees_
*R*_am_ (s m^−1^)	31.6	7.1	10.9	13.0	28.7	11.6
*R*_ah,Thom_ (s m^−1^)	47.3	15.6	20.4	23.9	46.2	21.8
*R*_ah,Choudhury_ (s m^−1^)	38.0	8.1	18.1	21.3	74.4	21.2
*R*_ah,Su_ (s m^−1^)	36.3	9.6	16.3	21.1	36.5	21.1
*R*_ac,Su_ (s m^−1^)	37.8	10.3	18.0	23.6	39.2	24.0
*R*_bh,Thom_ (s m^−1^)	15.6	8.5	9.4	10.9	17.7	10.0
*R*_bh,Choudhury_ (s m^−1^)	6.3	1.0	7.0	8.0	41.9	9.3
*R*_bh,Su_ (s m^−1^)	4.8	2.5	5.3	8.0	8.3	9.4
$kB_{h,\text{Thom}}^{-1}$	1.6	2.2	2.1	1.9	1.5	2.0
$kB_{h,\text{Choudhury}}^{-1}$	0.7	0.3	1.6	1.5	3.8	1.9
$kB_{h,\text{Su}}^{-1}$	0.5	0.7	1.2	1.5	0.7	1.9
Ω_Jarvis_	0.49	0.13	0.14	0.22	0.41	0.08
Ω_Martin_	0.38	0.10	0.12	0.19	0.35	0.07
$z_{0{\text{m}}_{\text{zh}}}$ (m)	0.05	2.65	0.55	0.80	0.02	0.80
$z_{0{\text{m}}_{\text{zh}\;\&\;\text{LAI}}}$ (m)	0.04	1.42	0.48	0.78	0.02	0.86
$z_{0{\text{m}}_{\text{wind}\;\text{profile}}}$ (m)	0.05	1.74	0.43	0.38	0.05	0.48
*ζ*	-0.021	-0.085	-0.034	-0.052	-0.030	-0.017
*L* (m)	-11.8	-137.1	-87.7	-62.7	-12.1	-153.2
*u*(*z*_h_)/*u*(*z*_r_)	0.29	0.62	0.48	0.60	0.02	0.56
*T*_s_ − *T*_a_(°C)	1.0	1.3	2.1	1.9	1.6	0.9
*T*_r_ − *T*_a_(°C)	0.2	0.5	0.9	2.5	2.7	2.6
*e*_s_ − *e*_a_ (kPa)	0.35	0.06	0.09	0.15	0.27	0.04
*D*_s_ − *D*_a_ (kPa)	-0.16	0.12	0.23	0.11	-0.03	0.09
*C*_s_ − *C*_a_ (μmol mol^−1^)	-13.8	-3.3	-2.8	-3.1	-4.7	-1.8
λ*E*_pot,PT_ (W m^−2^)	247.5	310.7	353.0	333.9	187.3	152.9
λ*E*_pot,PM_ (W m^−2^) ^a^	265.5	226.7	227.1	268.5	214.5	132.2
λ*E*_eq_ (W m^−2^)	196.4	246.6	280.1	265.0	148.6	121.3
λ*E*_imp_ (W m^−2^)	163.4	91.7	71.4	98.0	129.3	28.3
EBR	0.80	0.81	0.69	0.70	0.99	0.59
EB slope	0.72	0.76	0.64	0.67	0.98	0.43
EB intercept (W m^−2^)	21	18	19	10	3	27
Sp (W m^−2^)	6.2	5.5	2.8	2.3	2.0	1.3
EUE	0.038	0.022	0.012	0.012	0.021	0.008

^a^ with *G*_sw,ref_ taken as the 95% quantile of *G*_sw_

**Table 3:** Median daytime physiological ecosystem properties in the growing season calculated with the bigleaf package.

	AT-Neu	DE-Tha	FR-Pue	ES-LMa	ES-LMa_grass_	ES-LMa_trees_
WUE (g C (kg H_2_O)^−1^)	4.8	5.2	3.1	2.3	2.7	2.2
IWUE (g C kPa (kg H_2_O)^−1^)	5.3	5.4	4.2	3.1	3.4	3.1
uWUE (g C kPa^0.5^ (kg H_2_O)^−1^)	5.1	5.3	3.6	2.7	3.0	2.6
*G*_sw_ (mol m^−2^ s^−1^)	0.301	0.195	0.119	0.157	0.223	0.047
*m* (mol m^−2^ s^−1^ ln(kPa)^−1^)	0.080	0.091	0.089	0.099	0.067	0.060
*b* (mol m^−2^ s^−1^)	0.349	0.231	0.184	0.229	0.282	0.094
*G*_0,USO_ (mol m^−2^ s^−1^)	0.090	-0.007	-0.015	0.014	0.040	0.034
*G*_1,USO_ (kPa^0.5^) ^a^	1.4	1.5	2.3	3.5	4.1	2.9
*G*_1,BB_ ^a^	6.6	7.7	10.4	13.7	14.8	11.9
*G*_1,LEU_^a^^,^ ^b^	5.5	6.0	10.7	9.7	9.0	26.0
*D*_0_ (kPa)	1.7	1.5	0.9	2.1	4.7	0.3
*C*_i_ (μmol mol^−1^)	231	213	233	297	316	310
*C*_i_/*C*_s_	0.61	0.57	0.62	0.74	0.79	0.77
*V*_cmax,25_ (μmol m^−2^ s^−1^)	177.4	135.1	68.9	53.8	55.4	12.0
*J*_max,25_ (μmol m^−2^ s^−1^)	457.5	188.4	65.1	50.1	83.2	15.4
*α* (μmol CO_2_ m^−2^ s^−1^ (μmol quanta m^−2^ s^−1^)^−1^)	0.106	0.079	0.037	0.037	0.044	0.098
GPP_ref_ (μmol m^−2^ s^−1^) ^c^	34.8	24.0	12.9	12.5	13.8	8.6
LUE (mol mol^−1^)	0.027	0.020	0.010	0.008	0.009	0.002

^a^ assuming *g*_0_ = 0;

^b^ assuming *D*_0_ = 1.5 kPa;

^c^ at 2000 μmol m^−2^ s^−1^

## Discussion

### Potential and limitations of the ‘big-leaf’ approach

All calculations implemented in the bigleaf package are based on the ‘big-leaf’ framework [35, 44], which reduces the ecosystem to a single, uniform plane (Fig 1). This approach thus assumes that vegetation as well as meteorological conditions are vertically and horizontally homogenous. One advantage of the ‘big-leaf’ approach is that calculations require no additional information on the EC site or commonly available variables only (e.g. LAI, vegetation height). Ecosystem properties are inferred directly from EC measurements, with no assumptions on the underlying ecosystem structure. The ‘big-leaf’ approach is further applicable to both single-level and two-level EC systems. In the latter case ecosystem properties can be derived for two ‘big leafs’, e.g. whole ecosystem and understory [28, 29] or whole ecosystem and grass layer (this study, Fig 5).

It is important to clarify that the bigleaf package exclusively applies a top-down approach, in which the ‘big-leaf’ framework is used to estimate ecosystem properties inversely from the measured fluxes. The package does not provide bottom-up model formulations, which apply a ‘big-leaf’ framework to up-scale simulated fluxes from leaf- to canopy-level. This up-scaling approach has been shown to be prone to integration errors [31, 89]. However, this type of error does not apply to the calculations in the bigleaf package because the ‘big-leaf’ framework is solely used for the derivation of bulk ecosystem properties and no up- or down-scaling is performed.

The fact that the top-down ‘big-leaf’ approach as applied in this package can only derive bulk ecosystem properties is also its most critical limitation. It is not possible to resolve the vertical distribution of the derived properties. For example, soil and vegetation components cannot be distinguished and the resulting properties will inevitably contain signals from both the soil and the vegetation. These drawbacks can only be circumvented by modeling approaches such as two-layer (soil/canopy) [33, 51] or dual-source (sun/shade) models [31], which attempt to resolve the flux contribution of different canopy fractions or ecosystem compartments. These alternative modeling frameworks are more complex and consequently require additional site-specific information (e.g. canopy clumping, canopy nitrogen profiles, etc.). They are thus mostly applied to a few sites where these additional model parameters are sufficiently well known (e.g. [90, 91]). The ‘big-leaf’ framework is thus most suitable for multi-site comparisons or for sites where little ancillary information is available, and where no detailed knowledge on the derived variable (e.g. canopy gradients) is required.

### Interpretation of the derived physiological properties

The bigleaf package provides functions to calculate ecosystem-scale physiological variables such as *G*_s_, *G*_1_, *C*_i_, *V*_cmax_, *J*_max_, and GPP_ref_ in the same manner as it is commonly done at leaf-level. Important in this context is that the interpretation of these bulk canopy variables is not as straightforward as that of their leaf-level analogues (see also [23]). This is due to 1) conceptual uncertainties (as discussed above), and 2) the presence of confounding physical factors. For instance, the intensity of the before-mentioned mixing of soil and vegetation signals increases with a decrease of vegetation density (i.e. LAI) of the ecosystem. [36] for instance showed that *G*_c_ is substantially overestimated in ecosystems with an LAI less than approx. 2. This does not mean that the calculation of *G*_s_ is meaningless in low-LAI ecosystems, but its physiological interpretation as *G*_c_ is increasingly compromised as vegetation cover decreases. For ecosystems with an LAI lower than 2-3, the inversion of a soil/canopy model [33] is likely more appropriate than the inversion of the ‘big-leaf’ model for the derivation of physiological variables.

In all ecosystems, confounding physical factors, which are non-existent or negligible at leaf-level, must be taken into account in order to extract a meaningful physiological signal. For example, evaporation (i.e. water fluxes not under plant control) occurring after rainfall will lead to an overestimation of the stomatal slope parameter *G*_1_, and thus to an underestimation of WUE, if such time-periods are not filtered out (see [23] for an overview of confounding factors and their associated uncertainties).

In general, uncertainties of physiological variables propagate with each calculation step. For example, *C*_i_ as calculated by Eq 25 is affected by uncertainties in both input variables *G*_s_ and GPP. Photosynthetic parameters are affected by the same uncertainties and in addition by assumptions made for their calculation. It follows that with increasing number of calculation steps following the derivation of *G*_s_, uncertainties increase and the meaningfulness of the derived variables depends critically on the applied data filtering and the quality of the (original or partitioned) data.

As discussed above, all physiological variables are integrated over the entire canopy and represent bulk canopy properties (expressed in units per ground area instead of leaf area). They are thus not directly comparable to leaf-level measurements taken at a particular location in the canopy. The discrepancies between leaf and ecosystem values will be most pronounced for variables with a distinct profile within the canopy (e.g. *V*_cmax_ and *J*_max_ [31]), and probably less relevant for *G*_1_.

### General package usage guidelines

#### Data filtering

For most applications, it is recommended to apply a basic data filter that removes unreliable measurements or certain meteorological conditions. The optimal type of filter depends on the purpose of the study and the variable of interest. For example, it is advisable to exclude negative λ*E* values from the calculation of *G*_s_ in order to minimize the occurrence of negative *G*_s_ estimates which are not readily interpretable. Furthermore, periods outside the growing season or following rainfall should be removed if *G*_s_ is interpreted in an ecophysiological context. *G*_a_ and surface conditions on the other hand can in principle be calculated for all conditions. In general, data that do not fulfill the assumptions of the EC method, or that were gap-filled with low confidence, should be discarded. Depending on the filter settings and the conditions at the site, this can lead to a considerable fraction of missing values in the dataset. This is generally not a problem for the subsequent analyses in this package (missing input data simply return NA again), but some (regression-based) functions may require a minimum number of available data in order to return robust results.

#### Treatment of uncertainties

The derived variables in the bigleaf package are affected by several sources of uncertainty, which may be classified as 1) random errors in the measured fluxes [92, 93], 2) systematic errors in the fluxes due to e.g. energy-balance non-closure, advection problems [94, 95] and 3) conceptual uncertainties. The complex nature of uncertainties in EC measurements and the associated computational challenges to adequately account for and propagate all sources of uncertainty in the derived variables are the main reasons why the bigleaf package does not offer uncertainty estimates for each output interval. To account for one or more of the outlined sources of uncertainties, the use of wrapper functions is the most meaningful approach. These functions (often in specialized R packages) apply e.g. Monte Carlo (parameter sensitivity on the derived variables) or bootstrapping (random data sampling with replacement) techniques without the need to modify the functions in bigleaf. Some simple examples on the use of such wrapper functions are given in the vignette of the bigleaf package (accessible in R with browseVignettes(“bigleaf”).

#### Use of the derived properties

The majority of the derived properties in the bigleaf package are intended to be primarily diagnostic, i.e. results serve to provide a more mechanistic understanding of the observed fluxes, which enables a more comprehensive analysis and interpretation of ecosystem surface-atmosphere gas exchange. These diagnostics provide additional insights on the underlying physical or physiological processes and are often directly comparable across sites and climatic conditions. Some variables may further be helpful for the parameterization, calibration, or evaluation of bottom-up models. For that purpose, two major prerequisites must be fulfilled: (1) the variable of interest derived with a top-down (inversion) approach must be at the same organizational scale as the one calculated in the bottom-up model, and (2) the framework and the assumptions made in the two approaches must be consistent. For example, both the dynamics and magnitude of the simulated degree of atmosphere-canopy decoupling (Ω) by land surface models can be directly compared with the Ω values derived from this package [87]. This also applies to other characteristics such as *G*_a_, *G*_s_, or WUE and LUE metrics that are simulated as (emergent) bulk surface properties in models. In contrast, physiological bulk canopy parameters such as *C*_i_ should not be compared to leaf-level *c*_i_ values as simulated by multi-layer models. Likewise, bulk canopy or *V*_cmax,25_ cannot be used to parameterize leaf-level *v*_cmax,25_ in multi-layer models. In any case, it is imperative that uncertainties specific to the EC-method (as summarized in the previous section) are taken into account when derived properties are used for bottom-up modeling purposes.

## Conclusions

The presented R package bigleaf provides a framework for the derivation of physical and physiological ecosystem properties at EC sites in a consistent and reproducible manner and with minimal requirements regarding ancillary site data. The package thus has the potential to increase the comparability of the provided calculations as well as their applicability across sites. The functions will be useful in complementing the analysis of land-atmosphere mass and energy fluxes by providing a basic level of process understanding. The availability of additional ecosystem surface characteristics as provided by the bigleaf package will be key in interpreting ever-increasing records of EC data and the responses of land-atmosphere exchange to global environmental change. The open source and version control environment further enable the continuous development of the package and encourage community input.
